# Coronary Artery Stenosis and High-Risk Plaque Assessed With an Unsupervised Fully Automated Deep Learning Technique

**DOI:** 10.1016/j.jacadv.2024.100861

**Published:** 2024-03-06

**Authors:** Abdul Rahman Ihdayhid, Amro Sehly, Albert He, Jack Joyner, Julien Flack, John Konstantopoulos, David E. Newby, Michelle C. Williams, Brian S. Ko, Benjamin J.W. Chow, Girish Dwivedi

**Affiliations:** a Fiona Stanley Hospital, Perth, Australia; b Artrya Ltd, Perth, Australia; c Harry Perkins Institute of Medical Research, Perth, Australia; d Curtin University, Perth, Australia; e The University of Edinburgh, Edinburgh, Scotland; f Monash University, Melbourne, Australia; g University of Ottawa Heart Institute, Ottawa, Ontario, Canada; h University of Western Australia, Perth, Australia

**Keywords:** artificial intelligence, cardiac computed tomography, coronary artery disease, deep learning

## Abstract

**Background:**

Coronary computed tomography angiography (CCTA) has emerged as a reliable noninvasive modality to assess coronary artery stenosis and high-risk plaque (HRP). However, CCTA assessment of stenosis and HRP is time-consuming and requires specialized training, limiting its clinical translation.

**Objectives:**

The aim of this study is to develop and validate a fully automated deep learning system capable of characterizing stenosis severity and HRP on CCTA.

**Methods:**

A deep learning system was trained to assess stenosis and HRP on CCTA scans from 570 patients in multiple centers. Stenosis severity was categorized as >0%, 1 to 49%, ≥50%, and ≥70%. HRP was defined as low attenuation plaque (≤30 HU), positive remodeling (≥10% diameter), and spotty calcification (<3 mm). The model was then tested on 769 patients (3,012 vessels) for stenosis severity and 45 patients (325 vessels) for HRP.

**Results:**

Our deep learning system achieved 93.5% per-vessel agreement within 1 Coronary Artery Disease-Reporting and Data System (CAD-RADS) category for stenosis. Diagnostic performance for per-vessel stenosis was very good for sensitivity, specificity, positive predictive value, negative predictive value, and area under the curve with: >0% stenosis: 90.6%, 88.8%, 83.4%, 93.9%, 89.7%, respectively; ≥50% stenosis: 87.1%, 92.3%, 60.9%, 98.1%, 89.7%, respectively. Similarly, the per-vessel HRP feature achieved very good diagnostic performance with an area under the curve of 0.80, 0.79, and 0.77 for low attenuation plaque, spotty calcification, and positive remodeling, respectively.

**Conclusions:**

A fully automated unsupervised deep learning system can rapidly evaluate stenosis severity and characterize HRP with very good diagnostic performance on CCTA.

Coronary computed tomography angiography (CCTA) has an established role in the routine noninvasive assessment of coronary artery disease in patients with low- to intermediate-risk chest pain.^1^, ^2^, ^3^, ^4^ In addition to coronary artery stenosis, CCTA can reliably characterize the presence of high-risk plaque (HRP).^1^^,^^5^, ^6^, ^7^, ^8^ Despite the appeal of noninvasively quantified HRP features, the clinical translation of first-line CCTA is limited by its time-consuming manual reporting process as well as its dependence on specialized training and experience.^9^ The inaccurate manual reporting of CCTA can lead to inappropriate downstream investigations, such as invasive coronary angiography, incurring unnecessary healthcare costs.

Deep learning is a rapidly evolving field within artificial intelligence (AI) that has been applied to noninvasive imaging, demonstrating significant improvements in efficiency.^10^, ^11^, ^12^, ^13^, ^14^ This has led to the development of automated models for the assessment of coronary stenosis and HRP features.^15^^,^^16^ Despite promising results, further optimization and evidence demonstrating clinical utility are needed prior to wide-spread clinical acceptance. A fully automated deep learning approach to CCTA analysis that requires minimal human involvement would be more time-efficient, improve clinical translation, and reduce the clinical resources and workload required for comprehensive analysis compared with expert human readers. The aim of our study was to develop and validate a fully automated, ‘end-to-end’ deep learning system capable of rapidly evaluating stenosis severity and characterizing HRP features on CCTA.

## Methods

### Study design

We included patients aged ≥18 years with suspected coronary artery disease who underwent CCTA at 2 institutes based within Australia. We excluded scans with coronary stenting, coronary grafts, anomalous coronary arteries, or scans that were deemed nondiagnostic due to motion artifacts or image degradation. Furthermore, scans with nonoptimal opacification of the coronary vasculature as recommended by the Society of Cardiac Computed Tomography Guidelines (<250 HU or >1,000 HU) were excluded.^17^ All CCTA scans were reported by Level 3 expert readers with manual quantification of stenosis and HRP features. Patient demographics including age, gender, and Agatston coronary artery calcium score were recorded.

### CCTA acquisition

CCTA scans were performed on a 2 × 192-dual-source CT (DSCT) Siemens SOMATOM Force CT, a 2 × 128-DSCT Siemens SOMATOM Flash CT, a 2 × 128-DSCT Siemens SOMATOM Drive CT (Siemens Healthineers), and 160-slice Toshiba Aquilion ONE (Canon Medial Systems). Scan techniques included both prospective and retrospective acquisition using electrocardiogram gating based upon site protocols. Patients received beta blockade, nitroglycerin, and iodinated contrast in accordance with site protocols and Society of Cardiovascular Computed Tomography guidelines.^17^

### Model development

Scans were randomly divided into training (n = 313, 23.4%), validation (n = 257, 19.2%), and testing (n = 769, 57.4%) groups. A deep learning algorithm was developed that was comprised of a 3-dimensional (3D) convolutional neural network based on a U-Net architecture. The unsupervised deep learning algorithm was applied to automate the process of coronary vessel detection, centerline extraction, and vessel wall and lumen segmentation. Images were segmented on a voxel-level with contouring achieved of the vessel wall and lumen. The lumen was segmented from the straightened multiplanar reformation of the tracked coronary artery. Coronary centerlines are extracted by identifying possible coronary artery seed points and tracking and connecting individual seed points into a coronary tree. A classifier is used to label the extracted arteries based on a range of features. Following this, further deep learning algorithms determine the output of coronary stenosis and HRP. Discrepancies in the area between the lumen and the vessel wall were identified as regions of plaque, and stenosis was calculated using a healthy reference area to provide a maximal coronary stenosis output in the form of a percentage diameter stenosis value. Stenosis severity was categorized into 6 separate categories according to the Coronary Artery Disease-Reporting and Data System (CAD-RADS), at 0%, 1 to 24%, 25 to 49%, 50 to 69%, 70 to 99%, and 100%.^18^ For assessment of diagnostic performance, 4 categories of stenosis were analyzed: 0%, 1 to 49%, ≥50%, and ≥70%. Within regions of disease, the deep learning model was also able to characterize the presence of HRP consisting of low-attenuation plaque, positive remodeling, and spotty calcification. Low attenuation plaque was defined as a focal central area of plaque with an attenuation of <30 HU.^19^ Positive remodeling was defined as a remodeling index >1.1 by diameter when compared with a normal proximal vessel reference.^19^ Spotty calcification was defined as a small focal area of calcification measuring <3 mm in diameter surrounded by noncalcified plaque (plaque with an attenuation between 130 and 350 HU).^19^

### Training, validation, and testing

**Figure 1:**
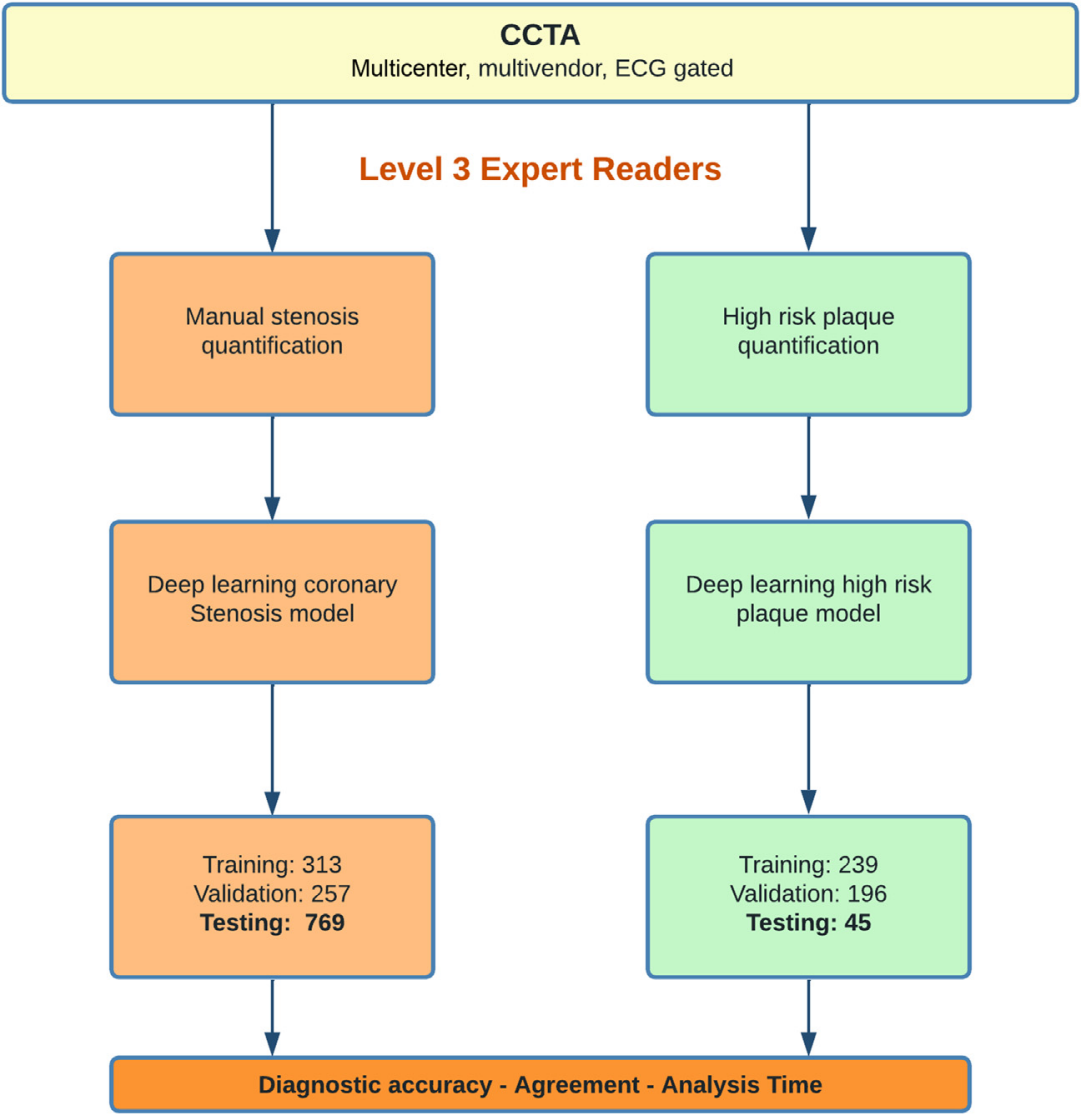
**Flow Chart Demonstrating the Number of Patients in the Training, Validation, and Testing Phases of the Deep Learning Model for Automated Detection of Coronary Artery Stenosis and High-Risk Plaque** This flow chart depicts the training, validation and testing for the deep learning system for both stenosis and high-risk plaque features. A total of 570 patients were used for training and validation for the coronary stenosis model; a subset of 435 patients were used to train and validate the high-risk plaque model. Finally, 769 patients were used for testing for the coronary stenosis model, with a subset of 45 patients used for testing the high-risk plaque model. CCTA = computerized coronary tomography angiography.

For training and validation of the deep learning system, CCTA scans from 570 patients were used from 4 separate institutions acquired with multivendor scanners. The vessels were manually segmented by level 3 expert annotators. All 570 patients were used for training and validation of the stenosis model, and a subset of 435 patients were used to train and validate the HRP model. The details on training and validation of the deep learning model are summarized in Figure 1. A multivendor testing dataset of scans from 769 patients (769 for stenosis and a subset of 45 for HRP) was used to assess the deep learning model’s diagnostic accuracy, agreement, and analysis time for assessment of stenosis and quantification of HRP.

### Level 3 expert reads

Each CCTA scan was read by at least 1 experienced reader with Level 3 (L3) qualifications, using semiautomatic commercially available software (TeraRecon CT Cardiac, TeraRecon Inc). A total of 4 readers were involved in the study. Each expert read represented ground truth for the corresponding study. Readers recorded CAD-RADS category, maximal percentage stenosis, and the qualitative visual presence (or absence) of HRP characteristics.

### Statistical analysis

Statistical analyses were performed using Python (Python Software Foundation). Data are presented as mean with standard deviation. Quadratic weighted kappa was used to measure agreement. Deep learning and L3 reads for maximal stenosis were compared on a per-patient and per-vessel basis. Diagnostic performance of deep learning vs L3 was assessed through diagnostic accuracy, sensitivity, specificity, positive predictive value, negative predictive value, and area under the receiver operating characteristic curve.

## Results

A total of 794 patients were used for testing of the model; scans from 25 patients were excluded due to poor image quality, image artifacts, anomalous coronary anatomy, or poor enhancement, leaving 769 patients in the final analysis (Supplemental Figure 1). Testing for stenosis detection was done on scans from all 769 patients, and a subset of scans from 45 patients underwent testing for HRP.

### Demographics and analysis time

The study population for the stenosis testing dataset comprised 769 patients (3,012 vessels) with a subset of 45 patients (325 vessels) for HRP characterization (Table 1). Demographics were similar across the stenosis and HRP groups, with a mean age of 62.5 ± 11.7 years and 61.5 ± 9.45 years in stenosis and HRP groups, respectively, and 64.4% male in the stenosis group and 76.1% male in the HRP group. Mean coronary artery calcium score was 393 ± 660 AU and 409 ± 536 AU for the stenosis and HRP groups, respectively.

**Table 1:** Demographic Characteristics of the Coronary Artery Stenosis and High-Risk Plaque Cohorts

	Coronary Artery Stenosis (n = 769)	High-Risk Plaque (n = 45)
Number of vessels	3,012	325
Age, y	62.5 ± 11.7	61.5 ± 9.45
Gender (male)	495 (64.4%)	35 (77.8%)
Agatston calcium score	392 ± 660	409 ± 536
Calcium score risk category		
0	183 (23.8%)	7 (15.5%)
1-10	67 (8.7%)	3 (6.7%)
11-100	134 (17.4%)	5 (11.1%)
101-400	132 (17.2%)	12 (26.7%)
>400	201 (26.1%)	14 (31.1%)
Unknown	52 (6.8%)	4 (8.9%)

Following CCTA input into the deep learning algorithm, the analysis times for the fully automated 3D convolutional neural network lumen and vessel wall segmentation and the combined deep learning-enhanced stenosis and HRP evaluation were 109.9 ± 43.4 and 29.7 ± 4.1 seconds, respectively.

### Coronary artery stenosis

**Figure 2:**
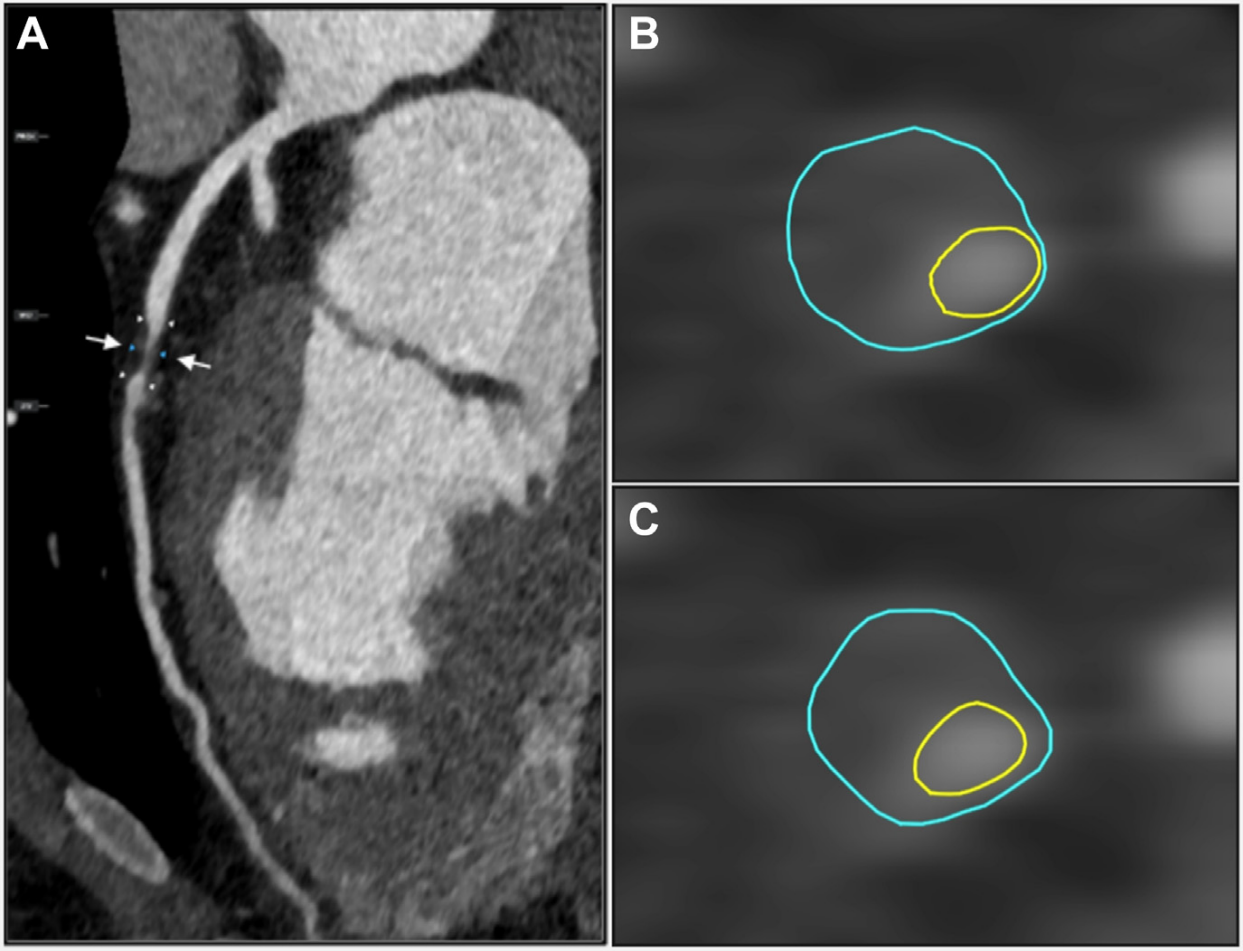
Comparison of Level 3 Expert Read and Deep Learning System Contouring of a Coronary Lesion (A) Depicts a lesion in the mid-segment of the left anterior descending artery. (B) Shows a cross-section through the lesion at the level of the white arrows with manual plaque analysis contours performed by level 3 expert read overlayed, displaying a severe (70%-99%) stenosis. (C) Shows the same cross-section with automated plaque and contouring by the deep learning system, where stenosis was also reported as severe (70%-99%).

Diagnostic performance of our deep learning model on a per-vessel and per-patient basis demonstrated very good specificity and negative predictive value for stenosis categories of 0%, 1 to 49%, ≥50% and ≥70% (Table 2, Figure 2). Overall, the misclassification rates for presence or absence of plaque were 10.5% and for the presence or absence of significant ≥70% coronary stenosis, 5.1%.

**Table 2:** Diagnostic Performance of the Deep Learning Model for Coronary Artery Stenosis on a Per-Vessel and Per-Patient Basis

Basis	Threshold	N	Sensitivity (95% CI)	Specificity (95% CI)	PPV (95% CI)	NPV (95% CI)	AUC (95% CI)	Accuracy (95% CI)
Per-vessel	>0%	1,151	91 (89-92)	89 (87-90)	83 (81-85)	94 (93-95)	90 (89-91)	90 (88-91)
	1-49%	788	68 (65-71)	91 (90-92)	73 (70-76)	89 (88-90)	80 (78-81)	85 (84-86)
	≥50%	363	87 (83-90)	92 (91-93)	61 (57-65)	98 (98-99)	90 (88-91)	92 (91-93)
	≥70%	129	59 (50-67)	97 (96-97)	43 (36-50)	98 (98-99)	78 (73-82)	95 (94-96)
Per-patient	>0%	526	95 (93-97)	75 (69-80)	89 (86-91)	88 (83-92)	85 (82-88)	89 (86-91)
	1-49%	259	66 (60-72)	89 (86-91)	75 (69-80)	84 (81-87)	78 (74-81)	81 (78-84)
	≥50%	267	94 (90-96)	83 (80-86)	75 (70-79)	96 (94-98)	89 (86-91)	87 (84-89)
	≥70%	111	60 (50-68)	90 (88-92)	51 (42-60)	93 (91-95)	75 (71-80)	86 (83-88)

AUC = area under the curve; NPV = negative predictive value; PPV = positive predictive value.

**Figure 3:**
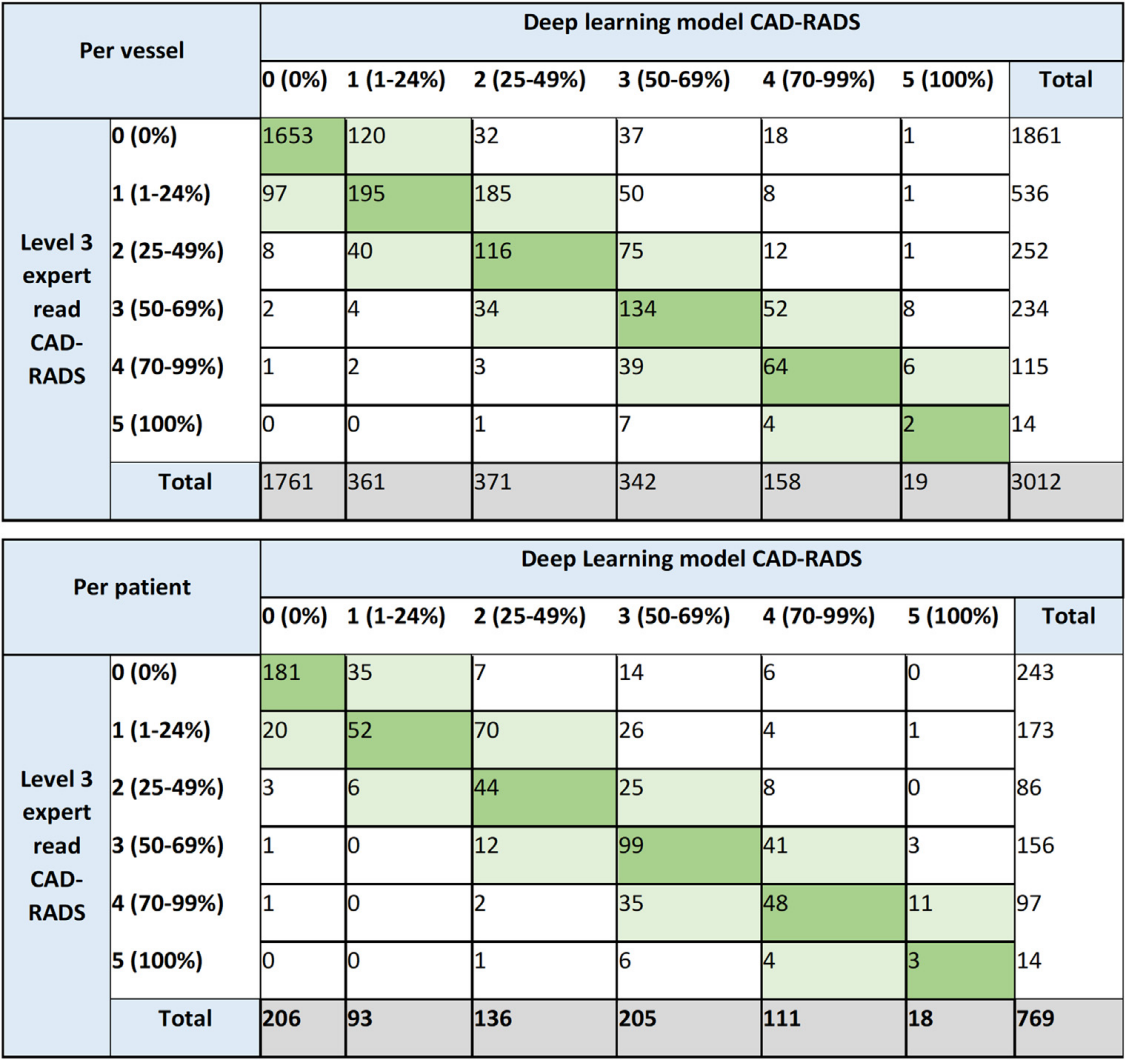
Level 3 Expert Read vs Deep Learning Model on a Per-Patient and Per-Vessel Basis Tables of level 3 expert read vs deep learning model CAD-RADS score on a per vessel (above) and per patient (below) basis. CAD-RADS = Coronary Artery Disease-Reporting and Data System.

Categorical CAD-RADS agreement between Level 3 expert readers and deep learning was 2,164/3,012 (71.8%) and 427/769 (55.5%) on a per-vessel and per-patient basis, respectively. Agreement within 1 CAD-RADS category was 2,816/3,012 (93.5%) and 686/769 (89.2%) on a per-vessel and per-patient basis, respectively (Figure 3). On a per-patient basis, the most frequent disagreement occurred with Level 3 expert reader CAD-RADS 1 and deep learning CAD-RADS 2 (n = 70; 40.5%). Based on current guidelines, further investigation is indicated with coronary artery stenosis ≥50% (CAD-RADS 3-5). The deep learning model demonstrated 93.6% and 87.1% accuracy for this group on a per-patient and per-vessel basis, respectively.^18^ Quadratic weighted kappa coefficient between Level 3 readers and deep learning was 0.78 and 0.79 per patient and per vessel, respectively.

### High-risk plaque characterization

HRP features were present in 29% (94/325) of the respective dataset as assessed by expert readers, including 13% (42/325) low attenuation plaque, 6% (19/325) positive remodeling, and 10% (33/325) spotty calcification. The deep learning system demonstrated high specificity (93.3%, 85.3%, and 85.6% for low attenuation plaque, positive remodeling, and spotty calcification, respectively), concordance (c-statistic 0.80, 0.77, and 0.79 for low attenuation plaque, positive remodeling, and spotty calcification, respectively) and diagnostic accuracy (90.0%, 84.3%, and 84.3% for low attenuation plaque, positive remodeling, and spotty calcification, respectively) at evaluating HRP when compared to Level 3 expert readers (Table 3).

**Table 3:** Diagnostic Performance of the Deep Learning Model for High-Risk Plaque Features on a Per-Vessel and Per-Patient Basis

Basis	High-Risk Plaque Feature	N	Sensitivity (95% CI)	Specificity (95% CI)	PPV (95% CI)	NPV (95% CI)	AUC (95% CI)	Accuracy (95% CI)
Per-vessel	Low-attenuation plaque	42	67 (52-79)	93 (90-96)	60 (45-72)	95 (92-97)	80 (72-87)	90 (86-93)
	Positive remodeling	19	68 (46-85)	85 (81-89)	22 (14-35)	98 (95-99)	77 (66-87)	84 (80-88)
	Spotty calcification	33	73 (56-85)	86 (81-89)	36 (26-48)	97 (94-98)	79 (71-87)	84 (80-88)
Per-patient	Low-attenuation plaque	29	79 (62-90)	81 (57-93)	89 (71-96)	68 (46-85)	80 (67-92)	80 (66-89)
	Positive remodeling	16	94 (72-99)	35 (20-53)	44 (29-61)	91 (62-98)	64 (54-74)	56 (41-69)
	Spotty calcification	23	91 (73-98)	46 (27-65)	64 (47-78)	83 (55-95)	68 (56-80)	69 (54-80)

AUC = area under the curve; NPV = negative predictive value; PPV = positive predictive value.

## Discussion

Our fully automated deep learning model demonstrated very good diagnostic accuracy and specificity in the quantification of coronary stenosis and HRP. This was unsupervised, automated, and completed rapidly, with an average total analysis time of <2.5 minutes per scan (Central Illustration). The inclusion of datasets from a range of different scanners is a key strength of this study, reflecting real-world clinical practice and optimizing the validity and applicability of the testing data results in a real-world population. This favorable result has important and encouraging implications for the future integration of AI-based cardiac CT analysis to assist expert readers in routine clinical practice.

**Central Illustration:**
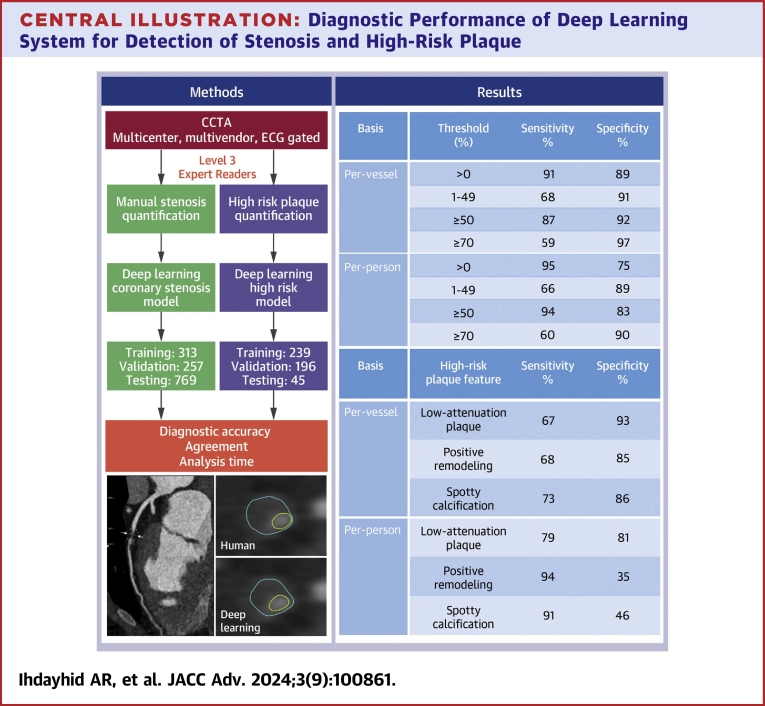
Diagnostic Performance of Deep Learning System for Detection of Stenosis and High-Risk Plaque A deep learning system was trained to assess coronary artery stenosis and high-risk plaque on cardiac computed tomography angiography (CCTA) scans from 570 patients in multiple centers (left). The model was then tested on 769 patients for coronary artery stenosis and 45 patients for high-risk plaque quantification. The model showed very good diagnostic performance for both per-vessel and per-patient analysis for coronary artery stenosis and high-risk plaque quantification (right). CCTA = cardiac computed tomography angiography; ECG = electrocardiogram.

### Potential benefits of AI-assisted CCTA interpretation

As per American and European guidelines, CCTA has emerged as the front-line noninvasive investigation for coronary artery disease.^20^^,^^21^ The progressive integration of CCTA into the clinical workup of chest pain has already resulted in increasing demand.^22^ This growth has placed major demands on current cardiac CT readers and may be limited by the extensive training required for future readers.^23^ Accurate CCTA interpretation of stenosis severity is essential to reduce unnecessary downstream testing such as cardiac functional assessment and invasive coronary angiography, increasing healthcare expenditure. AI-assisted CCTA interpretation is expected to support experienced cardiac computed tomography readers in their clinical practice and reduce the time for comprehensive analysis and clinical workload. This is especially evident by the rapid analysis time achieved by our deep learning model, which could potentially lead to substantial time savings compared to current CCTA read times by human operators.^24^ Once fully validated, deep learning models for coronary stenosis severity assessment and HRP characterization may also be used to expedite the training of healthcare professionals in interpreting CCTA in a clinical setting.

### Coronary artery stenosis severity evaluation using deep learning methods

The CAD-RADS scoring system is recommended for standardized reporting of coronary stenosis on CCTA and is widely used to guide clinical decision-making.^18^ Our deep learning model demonstrated good agreement with exact CAD-RADS category compared with L3 readers. Notably, the results demonstrate less variability than among human readers with contemporary CCTA studies reporting interobserver agreement values of 0.52 per vessel segment.^25^ These results favor the integrated use of deep learning in the assessment of coronary stenosis on CCTA. The strength of our deep learning system relates to its ability to not miss obstructive disease, as evidenced by the negative predictive value of 96.5%.

Our results and findings are consistent and similar with those presented by Choi et al, Lin et al, and Khasanova et al, achieving favorable correlation and kappa coefficients for AI tools compared to expert readers.^15^^,^^16^^,^^26^ Importantly, the deep learning technique utilized by Choi et al^15^ involved a quality-control cardiac CT-trained technician to review the AI analysis and make manual adjustment to the segmentation prior to finalizing the results. Lin et al^16^ required semiautomated coronary artery centerline extraction as a preprocessing step. Additionally, Khasanova et al^26^ had a human analyst verify and select an appropriate CCTA series for the algorithm and edit the segmentation output of the AI model. Comparatively, our deep learning system is unsupervised and fully automated, achieving these results without a ‘human-in-the-loop’. This unique feature may minimize user workload, enhance integration into the clinical workspace, and also reduce analysis time.

### High-risk plaque evaluation using deep learning methods

The identification of HRP is important as its presence has a strong association with fatal or nonfatal myocardial infarction in patients with stable chest pain.^6^^,^^8^^,^^27^, ^28^, ^29^, ^30^ CCTA is a reliable noninvasive modality for assessment of HRP; however, its clinical translation has been limited by the extensive training required for interpretation and the prolonged time taken for analysis.^8^^,^^24^^,^^27^, ^28^, ^29^, ^30^ A rapid deep learning model would help to improve clinical translation of CCTA-based assessment of HRP into routine clinical practice, enhancing cardiovascular risk stratification. The deep learning system developed by Lin et al^16^ demonstrated rapid and accurate quantification of plaque volume compared to expert readers. Our deep learning system is the first to effectively identify multiple HRP features of spotty calcification, positive remodeling, and low-attenuation plaque, which all have prognostic significance.^6^, ^7^, ^8^

Management options for HRP remain limited, with goal-directed medical therapy being the mainstay of treatment.^31^ This highlights the need to limit false positives in the detection of HRP, a strength of our deep learning model with specificities of 85.6%, 85.3%, and 93.3% for spotty calcification, positive remodeling, and low-attenuation plaque, respectively.

### Study Limitations

Although a total of 4 readers were involved in ground truth assessment, all CCTA scans were interpreted by a single reader. Therefore, consensus agreement and interobserver variability were not assessed in our study, potentially having an impact on true agreement and correlation between our deep learning model and ground truth. Furthermore, being a retrospective study, ground truth analysis was not validated against invasive methods of assessment including intravascular ultrasound or optical coherence tomography. Additionally, the sensitivity of our deep learning system was reduced in the ≥70% stenosis category, raising concerns of high false negatives in this group. However, most false negatives in this category remained in the ≥50% stenosis threshold (87% and 91% in per-vessel and per-patient analysis, respectively), prompting further investigation. Other limitations specific to the characterization of HRP relate to a modest sample size of 45 patients. We also excluded the napkin ring sign from our analysis due to its low prevalence in our testing dataset, which would negate any meaningful conclusions.

## Conclusions

We developed a fully automated, ‘end-to-end’, deep learning system that is capable of accurately and rapidly evaluating stenosis severity and characterizing HRP on CCTA, compared to Level 3 expert readers. Clinical translation of this algorithm as a supporting tool into real-world clinical practice has the potential to improve the clinical efficiency of performing CCTA on patients being investigated for coronary artery disease. Future research is needed to investigate the value and prognostic impact of incorporating deep learning techniques into clinical practice compared to standard of care.

PERSPECTIVES **COMPETENCY IN PATIENT CARE:** A fully automated deep learning-based system that can accurately and rapidly evaluate coronary artery stenosis and HRP characteristics on CCTA is feasible. This has the potential to improve the clinical efficiency of performing CCTA in patients being investigated for coronary artery disease.

**TRANSLATIONAL OUTLOOK:** Randomized trial data is necessary to validate this technology and evaluate the prognostic impact of incorporating it into clinical practice compared to standard care.

## Funding support and author disclosures

Drs Ihdayhid and Dwivedi are consultants and have equity interest in Artrya Ltd. Drs Joyner, Flack, and Konstantopoulos have equity interest in Artrya Ltd, Drs Joyner and Konstantopoulos are employees of Artrya Ltd. Dr Ihdayhid has consulting honorarium from Boston Scientific and Abbott Medical. Dr Williams (FS/ICRF/20/26002) is supported by the 10.13039/501100000274British Heart Foundation. Dr Williams has given talks for Canon Medical Systems, Siemens Healthineers, and Novartis. Dr Dwivedi has speaker bureau for Janssen, Amgen, Pfizer, and AstraZeneca. All other authors have reported that they have no relationships relevant to the contents of this paper to disclose.
