# Supplementary material for: Coronary Artery Stenosis and High-Risk Plaque Assessed With an Unsupervised Fully Automated Deep Learning Technique
Source: JACC Adv. 2024 Mar 6;3(9):100861. doi: 10.1016/j.jacadv.2024.100861 (PMC11450949; doi:10.1016/j.jacadv.2024.100861)
Supplement: Supplemental Figure 1 [file mmc1.pdf]

Supplementary Material

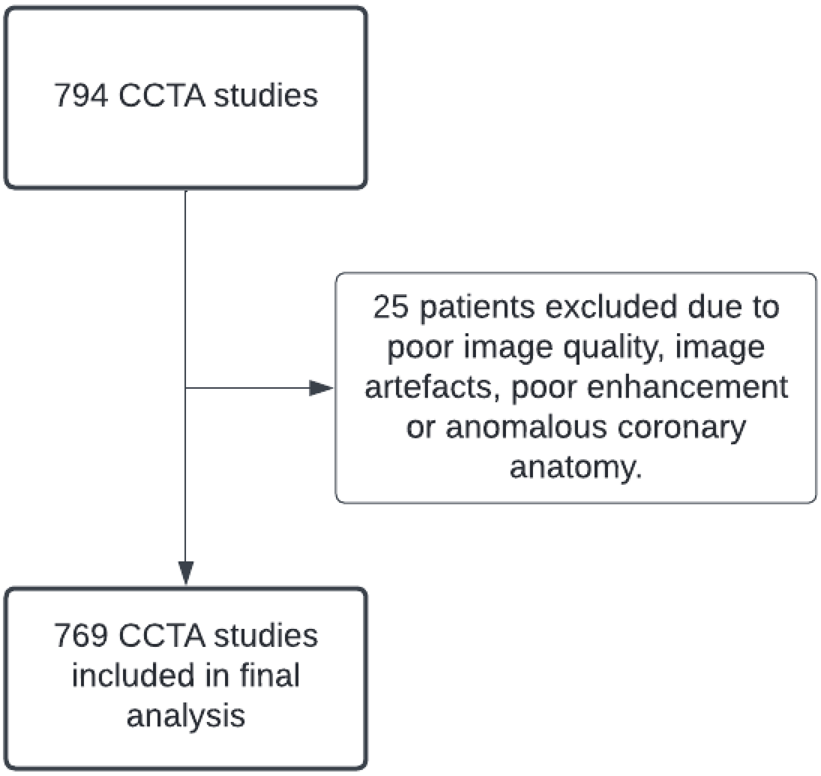

Supplementary Figure 1: Flow diagram of CCTA study inclusion from final analysis. Exams were excluded from analysis for poor image quality, artefact, poor enhancement, or anomalous coronary anatomy. Overall, 97% (769/794) were included in the final analysis.
